# Supplementary material for: Profile of Bacterial Community and Antibiotic Resistance Genes in Typical Vegetable Greenhouse Soil
Source: Int J Environ Res Public Health. 2022 Jun 24;19(13):7742. doi: 10.3390/ijerph19137742 (PMC9265268; doi:10.3390/ijerph19137742)
Supplement: Supplementary file 1 [file ijerph-19-07742-s001.zip › ijerph-1756985-supplementary.pdf]

Supplementary materials

1. Supplementary figure

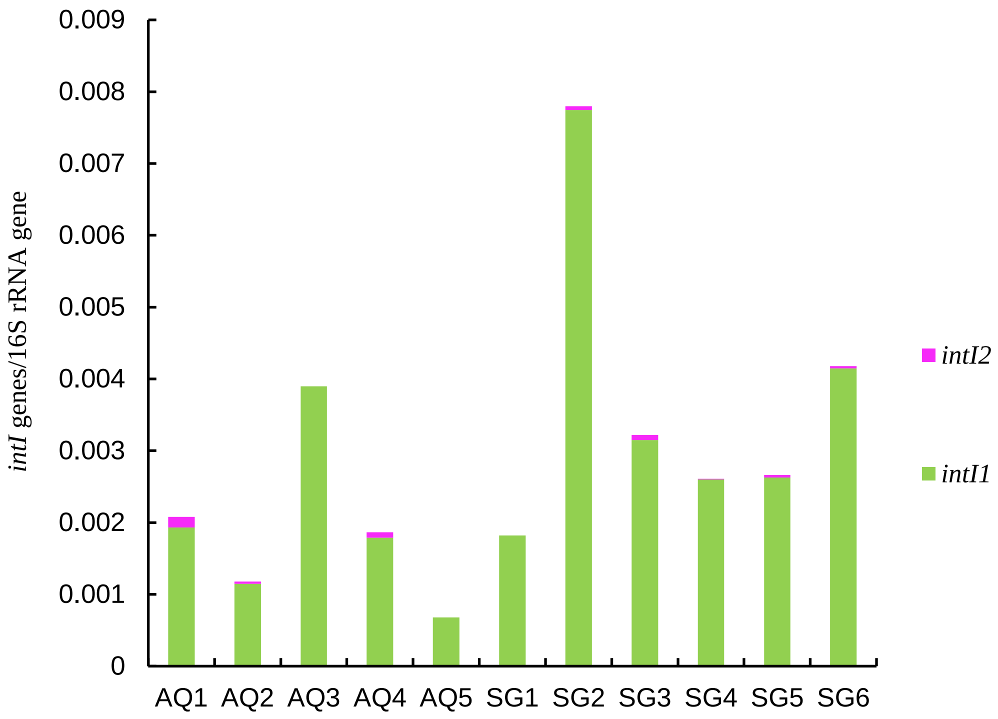

Figure S1 Relative abundances of *intI* genes in greenhouse soil

2. Supplementary tables

Table S1 Primers employed for qPCR

Genes	Forward oligonucleotide sequence (5′ to 3′)	Reverse oligonucleotide sequence (5′ to 3′)	Amplicon size (bp)	Annealing temperature (℃)	Reference
Integron-integrase genes					
intI1	GCCTTGATGTTACCCGAGAG	GATCGGTCGAATGCGTGT	196	60	[1]
intI2	TGCTTTTCCCACCCTTACC	GACGGCTACCCTCTGTTATCTC	195	60	[1]
β-lactam ARGs					
bla$_{TEM}$	GCKGCCAACTTACTTCTGACAACG	CTTTATCCGCCTCCATCCAGTCTA	247	55	[1]
Chloramphenicol ARGs					
cmlA	GCCAGCAGTGCCGTTTAT	GGCCACCTCCCAGTAGAA	158	55	[2]
Aminoglycoside ARGs					
aadA	GTGGATGGCGGCCTGAAGCC	AATGCCCAGTCGGCAGCG	528	54	[3]
aadA2	CGGTGACCATCGAAATTTCG	CTATAGCGCGGAGCGTCTCGC	250	54	[3]
strA	CCTGGTGATAACGGCAATTC	CCAATCGCAGATAGAAGGC	548	54	[3]
strB	ATCGTCAAGGGATTGAAACC	GGATCGTAGAACATATTGGC	509	54	[3]
Sulfonamide ARGs					
sul1	CACCGGAAACATCGCTGCA	AAGTTCCGCCGCAAGGCT	158	60	[1]
sul2	TCCGGTGGAGGCCGGTATATGG	CGGGAATGCCATCTGCCTTGAG	191	60.8	[1]
Tetracycline ARGs					
tetA	GCTACATCCTGCTTGCCTTC	CATAGATCGCCGTGAAGAGG	210	54	[3]
tetC	GCGGGATATCGTCCATTCCG	GCGTAGAGGATCCACAGGACG	207	68	[1]
tetG	GCTCGGTGGTATCTCTGCTC	AGCAACAGAATCGGGAACAC	468	55	[1]
tetB(P)	AAAACTTATTATATTATAGTG	TGGAGTATCAATAATATTCAC	169	46	[1]

tetL	GGTTTTGAAYGTYTCATTACCTGAT	GATAGCTTTCCATATASAGCTGTTCC	126	60	[1]
tetM	ACAGAAAGCTTATTATATAAC	TGGCGTGTCTATGATGTTCAC	171	55	[1]
tetO	TACGGARAGTTTATTGTATACC	TGGCGTATCTATAATGTTGAC	171	60	[1]
16S-rRNA	CAGCMGCCGCGGTAATWC	CCGTCAATTCMTTTRAGTTT	390	55	[1]

Table S2 The microbial α diversity index in vegetable greenhouse soil

**Soil**	**ACE**	**Chao1**	**Simpson**	**Shannon**
AQ1	1540±8	1567±12	0.024±0.003	5.77±0.06
AQ2	1473±18	1497±15	0.007±0.000	5.95±0.04
AQ3	1365±37	1388±47	0.009±0.001	5.79±0.04
AQ4	1500±17	1536±15	0.012±0.001	5.74±0.01
AQ5	1489±10	1518±25	0.008±0.000	5.88±0.02
SG1	1436±21	1454±18	0.016±0.002	5.65±0.04
SG2	1309±26	1325±42	0.009±0.000	5.63±0.01
SG3	1554±19	1579±31	0.009±0.000	5.93±0.01
SG4	1499±16	1537±22	0.009±0.001	5.79±0.02
SG5	1503±17	1546±34	0.011±0.000	5.75±0.02
SG6	1499±14	1511±19	0.013±0.002	5.62±0.07

Table S3 Physicochemical property and heavy metal concentration in vegetable greenhouse soil

Soil	As	Cu	Zn	Pb	Cd	Cr	Hg	Ni	Organic matter	pH
	$mg·kg^{-1}$	$mg·kg^{-1}$	$mg·kg^{-1}$	$mg·kg^{-1}$	$mg·kg^{-1}$	$mg·kg^{-1}$	$mg·kg^{-1}$	$mg·kg^{-1}$	%	
AQ1	6.50±0.11	34.22±1.26	35.83±1.11	23.74±1.29	0.13±0.01	85.90±3.33	0.048±0.002	40.38±2.35	4.73±0.16	6.14±0.14
AQ2	5.90±0.17	39.05±2.17	52.10±2.42	25.57±2.06	0.23±0.01	96.60±4.70	0.049±0.003	46.89±1.68	4.44±0.21	6.08±0.11
AQ3	6.88±0.22	42.68±2.36	44.83±2.25	25.88±2.16	0.17±0.01	101.00±6.85	0.057±0.003	41.46±2.78	3.27±0.18	6.20±0.17
AQ4	7.28±0.10	41.04±2.73	120.63±5.02	25.63±1.69	0.19±0.01	102.80±5.95	0.104±0.010	48.67±1.85	4.98±0.24	6.89±0.17
AQ5	5.76±0.09	19.05±0.90	66.13±2.61	24.58±1.98	0.30±0.01	108.00±5.24	0.070±0.003	45.08±1.65	4.18±0.17	6.86±0.23
SG1	6.87±0.16	17.57±1.14	34.13±2.22	20.65±2.29	0.11±0.01	63.50±1.61	0.062±0.001	25.80±0.92	2.44±0.25	7.37±0.15
SG2	6.40±0.25	12.99±1.64	42.57±2.98	22.17±1.05	0.12±0.01	64.95±2.35	0.066±0.003	28.09±1.29	2.93±0.19	6.88±0.29
SG3	6.53±0.23	16.12±1.08	48.33±1.86	22.33±1.36	0.12±0.01	65.37±2.78	0.072±0.003	27.84±1.02	2.33±0.18	7.19±0.16
SG4	5.69±0.29	27.33±1.50	141.30±4.91	22.36±1.76	0.24±0.02	86.24±3.38	0.043±0.003	25.81±2.30	5.24±0.20	6.13±0.22
SG5	4.63±0.21	27.55±1.65	119.23±6.66	23.29±2.06	0.24±0.02	67.70±1.77	0.083±0.004	28.19±2.14	4.60±0.18	6.43±0.16
SG6	3.73±0.26	46.40±1.13	170.87±7.40	21.91±2.26	0.25±0.02	58.50±3.09	0.044±0.002	25.74±1.83	5.81±0.15	6.46±0.16

1. Peng, S.; Feng, Y.; Wang, Y.; Guo, X.; Chu, H.; Lin, X., Prevalence of antibiotic resistance genes in soils after continually applied with different manure for 30 years. **2017,** 340, 16-25. https://doi.org/https://doi.org/10.1016/j.jhazmat.2017.06.059
2. He, L.; Ying, G.; Liu, Y.; Su, H.; Chen, J.; Liu, S.; Zhao, J., Discharge of swine wastes risks water quality and food safety: Antibiotics and antibiotic resistance genes from swine sources to the receiving environments. **2016,** 92-93, 210-219. https://doi.org/https://doi.org/10.1016/j.envint.2016.03.023
3. Pornsukarom, S.; Thakur, S., Horizontal dissemination of antimicrobial resistance determinants in multiple *Salmonella* serotypes following isolation from the environment of commercial swine operations after manure application. **2017**, AEM.01503-17. https://doi.org/https://doi.org/10.1128/AEM.01503-17
